# Clinical effect of successful reperfusion in patients presenting with NIHSS < 8: data from the BEYOND-SWIFT registry

**DOI:** 10.1007/s00415-018-09172-1

**Published:** 2019-01-08

**Authors:** Johannes Kaesmacher, Panagiotis Chaloulos-Iakovidis, Leonidas Panos, Pasquale Mordasini, Mirjam R. Heldner, Christoph C. Kurmann, Patrik Michel, Steven D. Hajdu, Marc Ribo, Manuel Requena, Christian Maegerlein, Benjamin Friedrich, Vincent Costalat, Amel Benali, Laurent Pierot, Matthias Gawlitza, Joanna Schaafsma, Vitor Mendes Pereira, Jan Gralla, Urs Fischer

**Affiliations:** 1 University Institute of Diagnostic and Interventional Neuroradiology, University Hospital Bern, Inselspital, University of Bern, Bern, Switzerland; 2 Department of Neurology, University Hospital Bern, Inselspital, University of Bern, Freiburgstrasse 8, 3010 Bern, Switzerland; 3 Department of Neurology, CHUV Lausanne, Lausanne, Switzerland; 4 Department of Radiology, CHUV Lausanne, Lausanne, Switzerland; 5 Department of Neurology, Vall d’Hebron University Hospital, Barcelona, Spain; 6 Department of Diagnostic and Interventional Neuroradiology, Klinikum rechts der Isar, Technical University Munich, Munich, Germany; 7 Department of Neuroradiology, CHU Montpellier, Montpellier, France; 8 Department of Neuroradiology, CHU Reims, Reims, France; 9 Department of Neurology, Toronto Western Hospital, Toronto, ON Canada; 10 Joint Department of Medical Imaging, Toronto Western Hospital, Toronto, ON Canada

**Keywords:** Mild symptoms, Mechanical thrombectomy, Endovascular, Low NIHSS, Thrombolysis, Stroke

## Abstract

**Background and purpose:**

If patients presenting with large vessel occlusions (LVO) and mild symptoms should be treated with endvoascular treatment (EVT) remains unclear. Aims of this study were (1) assessing the safety and technical efficacy of EVT in patients with NIHSS < 8 as opposed to a comparison group of patients presenting with NIHSS ≥ 8 and (2) evaluation of the clinical effect of reperfusion in patients with NIHSS < 8.

**Methods:**

Patients included into the retrospective multicenter BEYOND-SWIFT registry (NCT03496064) were analyzed. Clinical effect of achieving successful reperfusion (defined as modified Thrombolysis in Cerebral Infarction grade 2b/3) in patients presenting with NIHSS < 8 (*N* = 193) was evaluated using multivariable logistic regression analyses (displayed as adjusted Odds Ratios, aOR and 95% confidence intervals, 95%-CI). Primary outcome was excellent functional outcome (modified Rankin Scale, mRS 0–1) at day 90. Safety and efficacy of mechanical thrombectomy in patients with NIHSS < 8 was compared to patients presenting with NIHSS ≥ 8 (*N* = 1423).

**Results:**

Among patients with NIHSS < 8 (*N* = 193, 77/193, 39.9% receiving pre-interventional IV-tPA), successful reperfusion was significantly related to mRS 0–1 (aOR 3.217, 95%-CI 1.174–8.816) and reduced the chances of non-hemorrhagic neurological worsening (aOR 0.194, 95%-CI 0.050–0.756) after adjusting for prespecified confounders. In interaction analyses, the relative merits of achieving successful reperfusion were mostly comparable between patients presenting with NIHSS < 8 and NIHSS ≥ 8 as evidenced by non-significantly different aOR. Interventional safety and efficacy metrics were similar between patients with NIHSS < 8 and NIHSS ≥ 8.

**Conclusions:**

Achieving successful reperfusion is beneficial in patients with persisting LVO presenting with NIHSS < 8 and reduces the risk of non-hemorrhagic neurological worsening.

**Electronic supplementary material:**

The online version of this article (10.1007/s00415-018-09172-1) contains supplementary material, which is available to authorized users.

## Introduction

In a considerable proportion of stroke patients presenting with mild neurological symptoms, a proximal anterior circulation large-vessel occlusion (LVO) is identified as the underlying cause [1, 2]. However, in most patients with proximal LVO and low NIHSS scores on admission, indications for endovascular treatment (EVT) are not covered by current evidence derived from the large pivotal thrombectomy trials [3, 4]. In these patients, a well-developed collateral network presumably ensures sufficient blood flow to the territory distal to the occlusion site and neuronal functioning is largely maintained [5]. Intravenous thrombolysis (IVT) is the standard of care in patients presenting with mild disabling symptoms [6]. However, in patients with proximal LVO, IVT is often insufficient to recanalyze large clots and some patients deteriorate clinically [7–9] and tend to have poor outcomes if no reperfusion therapy is attempted, accordingly [10–12]. Hence, one of the most relevant unanswered questions is currently whether immediate EVT in patients with mild symptoms and LVO should be routinely considered to prevent clinical deterioration and to reduce infarct growth [11, 13–19]. While a proportion of patients will most likely benefit from routine EVT by preventing infarct growth, EVT also harbors the risk of worsening cerebral perfusion by thrombus dislocation and subsequent collateral shutdown and other procedural complications [20] (e.g. dissection [21], perforation [22], infarct to new territory [23], etc.).

The main aims of this registry analysis were (1) to assess safety and technical efficacy of EVT in LVO patients with NIHSS < 8 when opposed to a large comparison group of patients with severe symptoms and (2) to evaluate the clinical effect of timely blood flow restoration in patients with NIHSS < 8 as evidenced by measures of angiographical reperfusion success.

## Methods

### BEYOND-SWIFT registry

The Bernese–European RegistrY for ischemic stroke patients treated Outside current guidelines with Neurothrombectomy Devices using the SOLITAIRE™ FR With the Intention For Thrombectomy (BEYOND-SWIFT) is a retrospective, international, multicenter observational registry (https://clinicaltrials.gov/ct2/show/NCT03496064). Inclusion criteria were (1) Treatment with a Medtronic market-released neurothrombectomy device (applied as initial devices used for intervention) in acute ischemic stroke patients. Patients were treated at the discretion of the investigator, independent of participation in this registry; (2) Patients or patient’s legally authorized representatives have given informed consent according to Good Clinical Practices (GCP) and/or IRB and/or local or institutional policies allow use of observational registry data for research purposes.

Patients were excluded if they participated in another clinical trial or had withdrawn their consent for retrospective analyses. The following centers participated and contributed data of consecutive patients admitted to their hospital:

- Inselspital Bern, University Hospital Bern, University of Bern, Bern, Switzerland.
- CHUV, Lausanne University Hospital, Lausanne, Switzerland.
- Klinikum rechts der Isar, Technical University Munich, Munich, Germany.
- Montpellier CHU, University Hospital Montpellier, Montpellier, France.
- CHU Reims, University Hospital Reims, Reims, France.
- University Hospital Vall d’Hebron, Barcelona, Spain.
- Toronto Western Hospital—University Health Network, University of Toronto, Toronto, Canada.

An overview of included patients and rates of available follow-up data for each center can be found in Supplementary Table I. Ethical approval for inclusion of patient data in this pooled registry was obtained at each local responsible ethics committee (see Supplementary Table I). Additionally, ethical approval was obtained in Bern for pooling and anonymized analyses of the registry data (KEK Bern, Bern, Switzerland, Local Ethics Committee Study Identifier: 2018-00766).

**Fig. 1:**
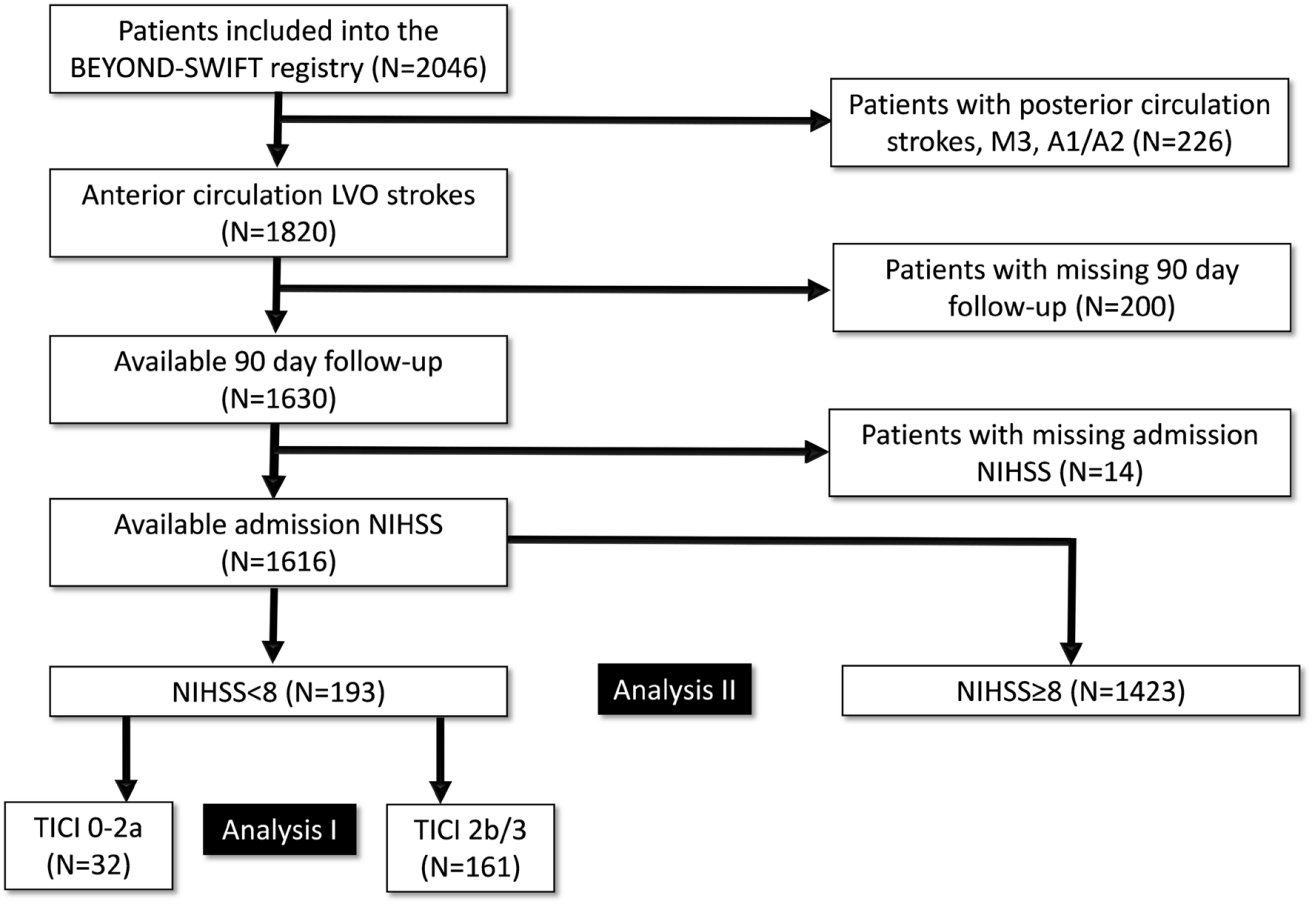
Study Flow Chart of the BEYOND-SWIFT Registry

Most patients included in the BEYOND-SWIFT (*n* = 2046) registry were treated for large-vessel anterior circulation strokes (*n* = 1820). Of these, 1630 had documented 90-day follow-up including 1616 with records of admission NIHSS. One-hundred-ninety-three of these patients presented with NIHSS < 8 (11.9%, see Fig. 1 for study flowchart). The corresponding lost-to-follow-up rate in the sub-group of patients with NIHSS < 8 was 6.3% (13/206).

### Variables and image analysis

The site of occlusion was categorized by local investigators into intracranial internal carotid artery, carotid-T/L, first/second/third segment of the middle cerebral artery (M1/M2/M3), first/second segment of the anterior cerebral artery (A1/A2), vertebral artery, basilar artery, or first/second segment of the posterior cerebral artery (P1/P2). For 7 patients, no data on occlusion site was available. Post-interventional modified thrombolysis in cerebral infarction (mTICI, 50% or more reperfusion defined as TICI2b) scale was operator adjudicated at each center or rated by an independent research fellow, depending on the standard of the respective centers (see Supplementary Table I) [24]. Anterior circulation extracranial–intracranial tandem occlusion were defined as the presence of an intracranial LVO and > 90% cervical stenosis or occlusion. Of 1616 patients with anterior circulation LVO strokes, records of admission NIHSS and day 90 follow-up,  974 (60.3%) underwent CT for admission workup, while 609 (37.7%) patients received magnetic resonance imaging (MRI) including diffusion-weighted sequences. For 33 patients  (2.0%) no information on admission imaging modality was provided. ASPECTS was evaluated at each site (see Supplementary Table I) and   scores were available for 1518/1616  (93.9%) patients in the whole cohort and in 184/193 (95.3%) patients with NIHSS<8 (including one patient with available ASPECTS but missing data on admission imaging modality). For clinical outcome evaluation, 3-month functional outcome was assessed applying the modified Rankin Scale (mRS) in routinely scheduled clinical visits or standardized telephone interviews, organized at each center. Rates of symptomatic intracerebral hemorrhage were reported by each center using the ECASS-II definition.

### Statistical analysis

The primary endpoint of this analysis was mRS 0–1 (excellent outcome) at day 90. Secondary and safety outcomes consisted of mRS 0–2 (good outcome) at day 90, all-cause mortality at day 90, non-hemorrhagic neurological worsening and symptomatic intracerebral hemorrhage, which was assessed at each center applying the ECASS II criteria. Non-hemorrhagic neurological worsening was defined as drop in the NIHSS ≥ 4 [25] between admission NIHSS and 24 h NIHSS without the occurrence of sICH [8]. Data on 24 h NIHSS were available for 1193/1616 in the complete cohort and 160/193 in the subcohort of patients with NIHSS < 8. Note that in a comparison of no non-hemorrhagic neurological worsening vs non-hemorrhagic neurological worsening, patients with sICH (*N* = 8) are excluded, leaving *N* = 152 patients for final analysis in the subcohort of patients with NIHSS <8. Confidence intervals of proportions were calculated using the method outlined by Wilson, implementing a continuity correction [26]. Univariate comparisons between patients in whom reperfusion was successful (mTICI 2b/3) and those in whom it was unsuccessful (mTICI ≤ 2a) were made using standard statistical measures (Fisher’s exact test for categorical variables, Whitney–Mann *U* Test for non-normally continuous or ordinally scaled variables and Welsch’s *t* test for independent normally distributed data). Association of successful reperfusion with all outcome parameters was assessed using multivariable logistic regression adjusting for the following pre-specified confounders: age (continuous), sex (categorical), NIHSS on admission (ordinal, adjusted odds ratio (aOR) per point increase), tandem vs non-tandem (categorical, tandem defined as > 90% cervical stenosis or cervical occlusion), center (categorical, contrast type: indicator, comparator: largest center), adjusted ASPECTS (see below, ordinal, adjusted odds ratio (aOR) per point increase), intravenous thrombolysis (categorical), risk factor hypertension (categorical), risk factor dyslipidemia (categorical), risk factor smoking (categorical), risk factor previous stroke (categorical), risk factor diabetes (categorical), in-hospital stroke (categorical), type of admission imaging (CT vs MRI, categorical), intracranial ICA/carotid-T vs M1 vs M2 occlusion (categorical, contrast type: indicator, comparator: ICA). To account for the imaging modality on admission (i.e. non-contrast CT vs DWI–MRI) on which the ASPECTS scoring was based upon, we increased all DWI–ASPECTS for regression analyses by 1 point according to the results derived from the SAMURAI registry [27]. For sensitivity purposes, analyses were rerun considering only patients with NIHSS < 6 and also after additional implementation of the interaction term successful reperfusion * IVT.

## Results

**Fig. 2:**
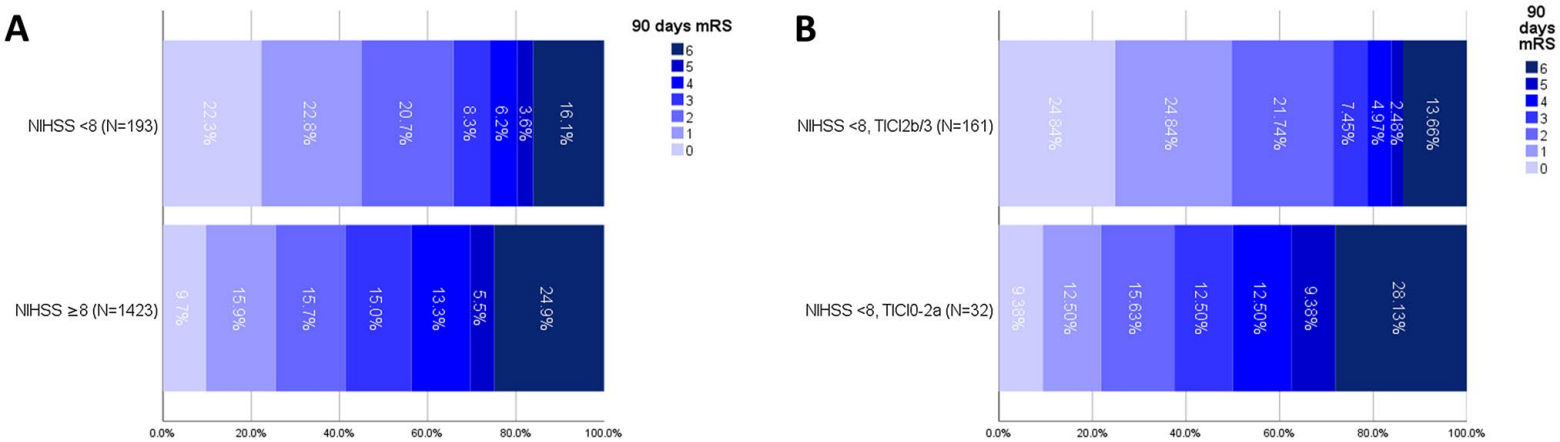
Day 90 Functional Outcome. **a** Comparison of patients with NIHSS < 8 and patients presenting with NIHSS ≥ 8; **b**, Patients with NIHSS < 8 dichotomized according to their reperfusion success; *NIHSS* National Institute of Health Stroke Scale, *mRS* modified Rankin Scale

Of the 1616 patients included into this analysis, 193 patients had an initial NIHSS < 8 (median NIHSS 5, IQR 4–6), comprising 103 patients with NIHSS < 6. Distribution of the NIHSS scores in treated patients with NIHSS < 8 is depicted in Supplementary Figure I. Patients with NIHSS < 8 had better outcome than patients with NIHSS ≥ 8 (*N* = 1423), as evidenced by higher rates of excellent functional outcome (mRS 0–1, 45.1% vs 25.6%, *P* < 0.001, see Fig. 2a) and lower mortality (16.1% vs 24.9%, *P* = 0.007). However, rates of non-hemorrhagic neurological worsening tended to be higher in patients with NIHSS < 8 as compared to patients with NIHSS ≥ 8 (13.8% vs 9.0%, *P* = 0.076).

In patients with NIHSS < 8, there were no differences in baseline characteristics, comorbidities or treatment metrics when comparing patients with and without successful reperfusion (Table 1). Admission ASPECTS were not different between patients with successful and without successful reperfusion (MRI-DWI: median 9 vs 8, p=0.235, CT: median 9 vs 9, 0.248, respectively). However, better outcomes were observed in patients with successful reperfusion as indicated by significantly higher rates of mRS 0–1 (49.7% vs 21.9%, *P* = 0.006, Fig. 2b) and mRS 0–2 (71.4% vs 37.5%, *P* < 0.001). Median NIHSS improvement at 24 h was 3 (IQR 0–5) in successfully reperfusion patients and − 3 in non-successfully reperfused patients (IQR − 11–0, *P* < 0.001, Supplementary Figure II). The overall incidence of non-hemorrhagic neurological worsening in patients without sICH was 13.8% (95%-CI 9.0%–20.6%), with significant differences among successfully reperfused patients and patients without successful reperfusion (9.9% vs 38.1%, *P* = 0.002). Rates of non-hemorrhagic neurological worsening were comparable among IVT and non-IVT pretreated patients (12.5% vs 14.8%, *P* = 0.813) and the reducing effect successful reperfusion on non-hemorrhagic neurological worsening was tangible also considering strata of IVT vs non-IVT patients (see Supplementary Table II). There was a trend towards lower mortality (13.7% vs 28.1%, *P* = 0.062) and lower rates of sICH (3.1% vs 9.4%, *P* = 0.131) if successful reperfusion was achieved.

**Table 1:** Patients with NIHSS < 8 stratified according to reperfusion success (TICI0-2a vs TICI2b/3)

NIHSS < 8, *N* = 193	TICI0-3 (*N* = 193)	TICI0-2a (*N* = 32)	TICI2b/3(*N* = 161)	*P* value
Age (years)	66.7 + /− 14.6	68.2 + /− 13.1	69.95 + /−14.9	0.503
Sex, female	55.4% (107/193)	53.1% (17/32)	55.9% (90/161)	0.846
Admission NIHSS	5 (IQR 4–6)	5 (IQR 3–6)	5 (IQR 4–6)	0.777
In-hospital stroke	1.6% (3)	0.0% (0/32)	1.9% (3/161)	> 0.999
Transfer	36.5% (70/192)	38.7% (12/31)	36.0% (58/161)	0.839
Witnessed symptom-onset/last-seen well to admission (min, *N* = 178)	148 (IQR 85–282)	201 (IQR 79–282, *N* = 29)	146 (IQR 87–285, *N* = 149)	0.769
Witnessed symptom-onset/last-seen well to groin puncture (min, *N* = 162)	257 (IQR 196–393)	295 (225–455, *N* = 26)	250 (195–370, *N* = 136)	0.248
Admission Imaging, MRI (*N* = 191)	57.6% (110/191)	50.0% (16/32)	59.1% (94/159)	0.433
IVT	39.9% (77/193)	31.3% (10/32)	41.6% (67/161)	0.326
*Risk factors*				
Smoking (N = 191)	28.8% (55/191)	18.8% (6/32)	30.8% (49/159)	0.203
Hypertension (N = 193)	60.1% (116/193)	56.3% (18/32)	60.9% (98/161)	0.694
Dyslipidemia (N = 192)	56.3% (108/192)	51.6% (16/31)	57.1% (92/161)	0.693
Previous CVE (N = 191)	12.6% (24/191)	3.1% (1/32)	14.5% (23/159)	0.086
Diabetes (N = 193)	13.5% (26/193)	21.9% (7/32)	11.8% (19/161)	0.154
Occlusion site				0.203
iICA	2.1% (4/193)	3.1% (1/32)	1.9% (3/161)	
Carotid-T/L	6.2% (12/193)	12.5% (4/32)	5.0% (8/161)	
*M*1	58.5% (113/193)	46.9% (15/32)	60.9% (98/161)	
*M*2	33.2% (64/193)	37.5% (12/32)	32.3% (52/161)	
Extracranial–intracranial tandem occlusion	15.5% (30/193)	21.9% (7/32)	14.3% (23/161)	0.290
Underlying cervical dissection	5.2% (10/193)	6.3% (2/32)	5.0% (8/161)	0.673
TOAST (*N* = 192)				0.242
Large-artery	9.4% (18/192)	15.6% (5/32)	8.1% (13/160)	
Cardioembolism	41.1% (79/192)	28.1% (9/32)	43.8% (70/160)	
Other	8.9% (17/192)	12.5% (4/32)	8.1% (13/160)	
Unknown	40.6% (78/192)	43.8% (14/32)	50.0% (64/160)	
*Outcome*				
mRS 0–1	45.1% (87/193)	21.9% (7/32)	49.7% (80/161)	0.006†
mRS 0–2	65.8% (127/193)	37.5% (12/32)	71.4% (115/161)	< 0.001†
Non-hemorrhagic neurological worsening (*N* = 152)	13.8% (21/152)	38.1% (8/21)	9.9% (13/131)	0.002†
Mortality	16.1% (31/193)	28.1% (9/32)	13.7% (22/161)	0.062
sICH (*N* = 192)	4.2% (8/192)	9.4% (3/32)	3.1% (5/160)	0.131

*NIHSS* National Institute of Health Stroke Scale, *TICI* Thrombolysis in Cerebral Infarction, *IVT* intravenous thrombolysis, *CVE* cerebrovascular event, *iICA* intracranial ICA, *TOAST* Trial of ORG 10,172 in Acute Stroke Treatment, *mRS* modified Rankin Scale, *IQR* interquartile range

^†^*P* < 0.01

**Fig. 3:**
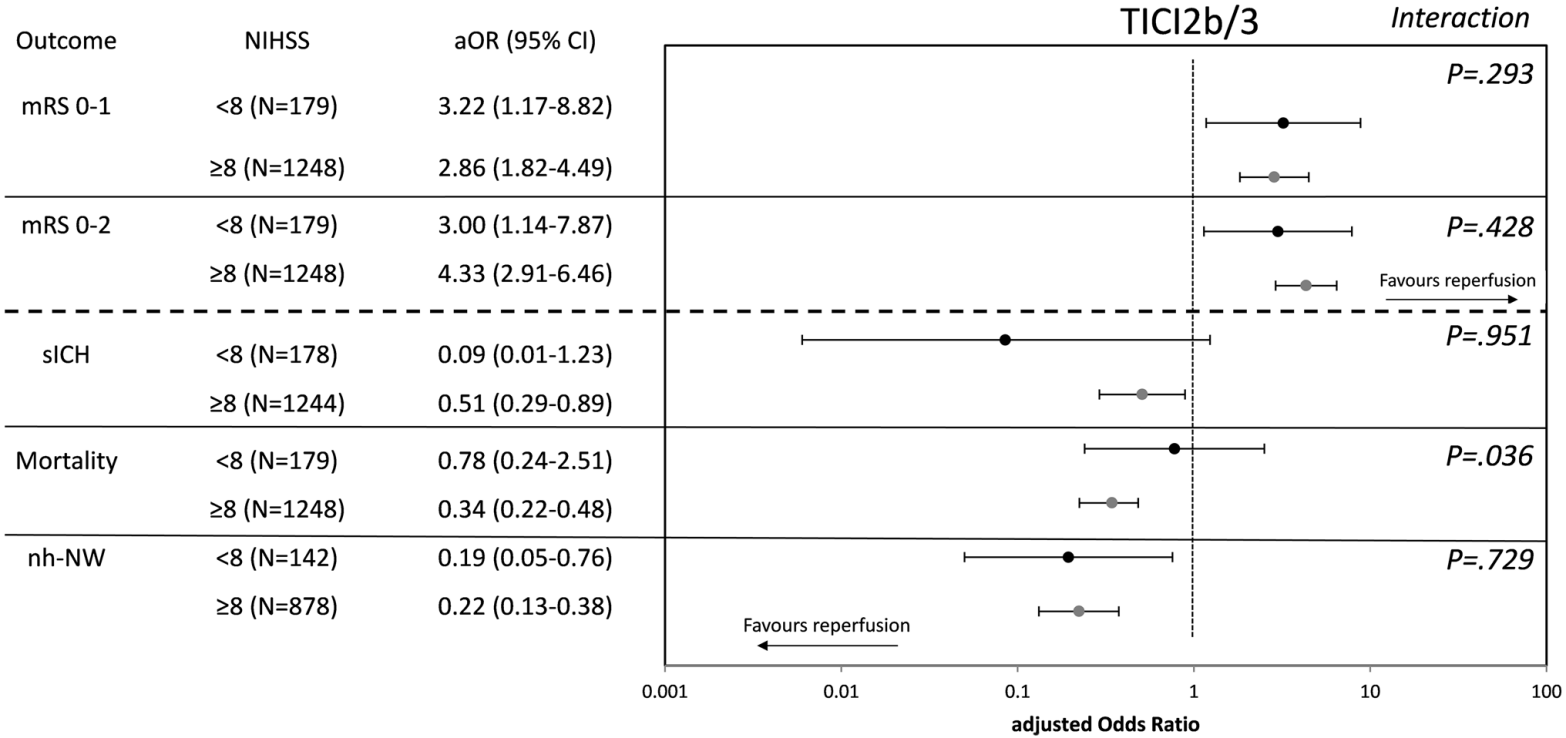
adjusted Odds Ratios of successful reperfusion (TICI2b/3) for various endpoints with strata of admission NIHSS < 8 vs NIHSS ≥ 8. Adjusted Odds Ratios were calculated in split cohorts using multivariable binary logistic regression adjusting for all variables outlined in the methods section. Analysis was rerun implementing the variable NIHSS < 8 vs NIHSS ≥ 8 and the term NIHSS < 8 vs NIHSS ≥ 8 * TICI2b/3 (reperfusion) to test for potential interaction in the whole cohort (the variable admission NIHSS was ommitted in this model, accordingly)

Among patients with NIHSS < 8, successful reperfusion was a significant factor related to mRS 0–1 (aOR 3.217, 95%-CI 1.174–8.816, Table 2), mRS 0–2 (aOR 2.995, 95%-CI 1.140–7.868) and reduced the chances of non-hemorrhagic neurological worsening (aOR 0.194, 95%-CI 0.050–0.756) in multivariable binary logistic regression analysis adjusting for prespecified confounders outlined in the methods section. No significant associations were found for the endpoints sICH (aOR 0.086, 95%-CI 0.006–1.234) and day 90 mortality (aOR 0.776, 95%-CI 0.240–2.509), although point estimates favored reperfusion. No significant interactions between the above-mentioned associations and IVT pretreatment status were observed (*P* for interaction > 0.05, see Table 2). The point estimates remained significant for mRS 0–1 in a subgroup of patients with NIHSS < 6, although uncertainty of the point estimates increased considerably (see Table 3). The relative merits of achieving successful reperfusion for various endpoints were comparable between patients presenting with NIHSS < 8 and NIHSS ≥ 8 (Fig. 3). However, reduction of mortality and sICH was found to be significant only in the cohort of patients presenting with NIHSS ≥ 8. Here, a significant interaction regarding the mortality reducing effect of successful reperfusion was found, suggesting the effect to be larger in patients presenting with NIHSS ≥ 8 (Fig. 3).

**Table 2:** Adjusted Odds ratios for TICI2b/3 for different outcome measures in patients with NIHSS < 8

NIHSS < 8 *N* = 193	*N* included in the model	aOR TICI2b/3	95%-CI	*P*	*P* for interaction with IVT
Primary					
mRS 0–1	179/193	3.217	1.174–8.816	0.023^†^	0.343
Secondary					
mRS 0–2	179/193	2.995	1.140–7.868	0.026^†^	0.430
sICH	178/193	0.086	0.006–1.234	0.071	0.997
Mortality	179/193	0.776	0.240–2.509	0.672	0.874
Non-hemorrhagic neurological worsening	142/193	0.194	0.050–0.756	0.018^†^	0.944

*NIHSS* National Institute of Health Stroke Scale, *aOR* adjusted Odds Ratio, *TICI* Thrombolysis in Cerebral Infarction, *95% CI* 95% confidence interval, *IVT* intravenous thrombolysis, *mRS* modified Rankin Scale, *sICH* symptomatic intracerebral hemorrhage

^†^*P* < 0.05

**Table 3:** Adjusted Odds ratios for TICI2b/3 for different outcome measures in patients with NIHSS < 6

NIHSS < 6 *N* = 103	*N* included in the model	aOR TICI2b/3	95%-CI	*P*	*P* for interaction with IVT
Primary					
mRS 0–1	98/103	4.878	1.196–19.889	0.027†	0.934
Secondary					
mRS 0–2	98/103	3.690	0.911–14.949	0.067	0.795
sICH	97/103	Did not converge	–	–	–
Mortality	98/103	1.068	0.180–6.343	0.942	0.936
Non-hemorrhagic neurological worsening	77/103	0.111	0.011–1.083	0.059	0.149

*NIHSS* National Institute of Health Stroke Scale, *aOR* adjusted Odds Ratio, *TICI* Thrombolysis in Cerebral Infarction, *95% CI* 95% confidence interval, *IVT* intravenous thrombolysis, *mRS* modified Rankin Scale, *sICH* symptomatic intracerebral hemorrhage

^†^*P* < 0.05

When comparing patients with NIHSS < 8 and patients with NIHSS ≥ 8, a tendency for shorter groin-puncture to reperfusion intervals in patients with NIHSS < 8 could be noted (median 42 min vs 47 min, *P* = 0.075). Apart from this, no differences in interventional safety and efficacy parameters were found (see Table 4). In particular rates of successful reperfusion (83.4% vs 82.2%, *P* = 0.690), TICI3 reperfusion (47.7% vs 44.4%, *P* = 0.441) and complications (11.4% vs 12.6%, *P* = 0.818) did not differ between patients presenting with mild symptoms and patients with NIHSS ≥ 8.

**Table 4:** Comparison of technical efficacy and safety in patients with NIHSS < 8 compared to patients with NIHSS ≥ 8

All anterior circulation LVO strokes with available mRS and NIHSS (*N* = 1621)	NIHSS < 8 (*N* = 193)	NIHSS ≥ 8 (*N* = 1423)	*P* Value
Successful reperfusion	83.4% (161/193)	82.2% (1169/1422)	0.690
TICI3	47.7% (92/193)	44.4% (633/1422)	0.441
Number of maneuvers (*N* = 1147)	1 (IQR 1–2, *N* = 158)	2 (IQR 1–3, *N* = 984)	0.129
Time from groin puncture to reperfusion (min, *N*=1507)	42 (IQR 29–65, *N* = 184)	47 (IQR 30–78, *N* = 1323)	0.075
Other devices used as rescue (*N* = 1338)	8.5% (14/164)	12.1% (142/1169)	0.196
Complications	11.4% (23/193)	12.6% (179/1420)	0.818
Type of complications (relative frequency)			
Vasospasms	30.4% (7/23)	24.7% (44/178)	
Dissection	34.8% (8/23)	16.9% (30/178)	
Perforation	13.0% (3/23)	11.8% (21/178)	
Other	13.0% (3/23)	14.6% (26/178)	
ENT	8.7% (2/23)	30.9% (55/178)	
Multiple	0% (0/23)	1.1% (2/178)	
Missing information on type of complication	–	*N* = 1	
sICH	4.2% (8/192)	6.0% (85/1417)	0.409

*NIHSS* National Institute of Health Stroke Scale, *mRS* modified Rankin Scale, *TICI* Thrombolysis in Cerebral Infarction, *ENT* emboli to new territory, *sICH* symptomatic intracerebral hemorrhage

## Discussion

This registry-based retrospective analysis of consecutive patients treated with stent-retrievers has the following main findings: (1) Successful reperfusion is associated with better functional long-term outcome and early neurologic recovery in treated patients presenting with NIHSS < 8. (2) The effect remained tangible in the subgroup of patients with NIHSS < 6, however, there is large uncertainty according to the small sample size. (3) When compared with patients presenting with severe symptoms, interventional procedures in patients with NIHSS < 8 were equally safe and technically effective and the relative merit of successful reperfusion on promoting good outcome was comparable.

Irrespective of treatment modalities, patients with low NIHSS and large-vessel occlusion have a more benign course than their counterparts presenting with severe neurological deficits [28]. Several underlying factors are causal for this observation, including incomplete occlusion [29, 30], partially permeable thrombi [31], and excellent collaterals, all of which allow for an adequately maintained blood flow to the brain tissue located distally to the LVO. However, there is compelling evidence that reperfusion therapies (IVT and/or EVT) improve the outcome of LVO patients also if they present only with mild neurological symptoms [11, 32–34]. Still, around 10–30% of patients will deteriorate after IVT treatment, which is a phenomenon related to lysis-refractoriness, “collateral-failure”[35–39] and thrombus extension [40]. Correspondingly, nearly one-quarter of patients primarily treated with medical therapy do not achieve functional independence [33] and > 60% of patients without successful reperfusion remained functionally depend in the presented cohort. As long as those patients—likely to deteriorate—cannot precisely be identified a priori, there are two general treatment approaches, both having their advantages and disadvantages: (1) Subjecting all patients with acute LVO to EVT, thus putting patients at interventional risk, who would have reperfused spontaneously or after IVT treatment at comparable time points; (2) Only subject patients to EVT if they experience clinical deterioration, implying that reperfusion will be achieved later and risking that additional tissue will undergo infarction. A recent multi-center study comparing both of these treatment approaches on a center-level basis, found that patients undergoing either emergent mechanical EVT or delayed EVT in case of secondary clinical deterioration had comparable outcomes [28]. However, depending on the type of analysis, a trend towards better outcome in patients undergoing emergent EVT was noted [28]. A similar observation was made in a multi-center cohort described by Haussen et al. Here, the authors found that mechanical thrombectomy in patients presenting with NIHSS ≤ 5 was associated with higher rates of functional independence and a favorable NIHSS shift when compared to medical management alone [34]. These findings were corroborated by a recent meta-analysis suggesting improved outcome in patients with NIHSS ≤ 8 if treated with thrombectomy as opposed to best medical treatment [41]. However, in this analysis, increased rates of sICH after endovascular therapy were found, warranting further data [41]. Importantly, in the presented cohort, the rates of sICH were markedly lower (4.2%) than the proportion of patients experiencing sICH described in the meta-analysis (13.6%) [41].

In line with our findings, several other single-arm EVT studies also found high rates of procedural success and good safety profiles of EVT in patients presenting with LVO and minor symptoms [18, 19, 42–44]. Corroborating our observations regarding the beneficial effect of successful reperfusion, Dargazanli et al. also reported that patients in whom TICI2b or TICI3 could be achieved do significantly better, although uncertainty of point estimates was relatively large [18]. In addition to these results, we found no evidence that the effect of achieving successful reperfusion is different in patients treated with IVT and those with contraindication for IVT. While the presented data is insufficient to answer the questions regarding preferred treatment regimens in the subgroup of patients presenting with low NIHSS, the data suggest that routine EVT in this subgroup of patients may be beneficial, because it is very likely that successful and timely reperfusion will be achieved more often.

The rate of complications in patients with NIHSS < 8 was comparable to recent meta-analysis data [20] and matches the frequency of complications observed in patients presenting with NIHSS ≥ 8 in this registry. Two-thirds of complications were comprised by iatrogenic cervical dissections and periprocedural vasospasms, both associated with relatively low risk of prompting unfavorable outcomes [21]. When adjusting for the lower rates of successful reperfusion in patients with procedural complications, we did not observe an independent effect of complications on outcome (data not shown). This suggests that not achieving successful reperfusion, rather than the complication itself puts patients at risk for poor outcomes. Moreover, some reassurance is provided that the rates of non-hemorrhagic neurological worsening are unlikely to be higher than if treated with IVT only. In a recent analysis of the SITS registry dealing with minor strokes treated with IVT, non-hemorrhagic neurological worsening was found in 30% of ICA-T or Tandem occlusions, 16.7% of other ICA occlusion, 9.3% of M1 and 5.8% of M2 occlusions [8]. Transferring those frequencies to our study population with available 24 h NIHSS, a rate of 11.9% could be expected (Supplementary Table III), which is non-significantly less frequent than what we have observed in our cohort (13.8%). It has to be kept in mind though, that early responders to IVT (unlikely to experience non-hemorrhagic neurological worsening) are a priori excluded in our cohort. Although the numbers were generally small, it should be stressed that more than every third patient with persistent occlusion on angiography in whom no successful reperfusion could be achieved experienced non-hemorrhagic neurological worsening in our cohort (38.1%, 8/21). Nevertheless, it seems obvious that subjecting all patients with NIHSS < 8 to EVT as a clinical routine will unavoidably put a minority of patients at risk for worsening of the perfusion status as compared to when endovascular treatment would have been withheld and our observation that non-hemorrhagic neurological worsening occurs more often in patients with low NIHSS supports this. Hence, it will need a randomized controlled trial to clarify if the potential benefits of preventing natural course deterioration outweighs the risks associated with procedural complications in patients who would otherwise stay clinically stable. Such a trial may also incorporate imaging selection criteria to identify patients with mild symptoms most likely to benefit [45]. In summary, the data presented stress that the effect of achieving successful reperfusion in the subgroup of patients with low NIHSS seems substantial and the comparable frequency of non-hemorrhagic neurological worsening between our cohort and findings derived from minor strokes patients included in the SITS registry further points towards safety equipoise in this subgroup of patients.

## Limitations

This is a single-arm multicenter retrospective registry and has associated limitations. No comparison to patients treated with medical management only was performed. Several factors including final mTICI score and initial ASPECTS were not core-lab adjudicated but were rated at the respective centers. Subgroup analyses were generally confined to small cohorts, which introduces a large uncertainty of the presented effects as indicated by relatively wide confidence intervals. Moreover, interaction analyses may be underpowered. The available sample represents minor strokes with LVO in whom the exact reasons for this treatment decisions are not known and may thus do not present the whole population of minor strokes with LVO. Importantly, patients who reperfused before endovascular treatment were excluded from the registry. Hence, interaction analysis or comparisons of e.g. bridging vs direct mechanical thrombectomy patients will only cover patients, who had persistent occlusions on the first angiography run, or did not clinically improve after IVT, respectively. The lack of a significant difference or interaction thus does not imply that pre-interventional IVT is not beneficial in this subcohort of patients, because at least 10%–20% of patients will reperfuse before endovascular thrombectomy [46, 47], which has been associated with better clinical outcomes.

## Conclusion

Achieving successful reperfusion in patients with persistent LVO on first angiography runs is beneficial in patients presenting with NIHSS < 8 and this effect is independent of whether patients were pretreated with IVT or not. Given the differences of the likelihood to achieve timely and complete reperfusion between best medical management and EVT, the presented data may be interpreted as a hint towards potential benefits of emergent routine EVT in this subgroup of patients. Randomized controlled trials comparing best medical treatment vs EVT in LVO patients with minor symptoms are warranted.

## Electronic supplementary material

Below is the link to the electronic supplementary material.

Supplementary material 1 (PDF 326 KB)
