# Supplementary material for: Clinical effect of successful reperfusion in patients presenting with NIHSS < 8: data from the BEYOND-SWIFT registry
Source: J Neurol. 2019 Jan 8;266(3):598–608. doi: 10.1007/s00415-018-09172-1 (PMC6394689; doi:10.1007/s00415-018-09172-1)
Supplement: Supplementary file 1 — Supplementary material 1 (PDF 326 KB) [file 415_2018_9172_MOESM1_ESM.pdf]

Clinical effect of successful reperfusion in patients presenting with NIHSS <8: Data from a multicenter registry.

Running title: Thrombectomy in patients with NIHSS <8

Johannes Kaesmacher MD[a,b], Panagiotis Chaloulos-Iakovidis MD[b], Leonidas Panos MD[b], Pasquale Mordasini MD[b], Mirjam R Heldner MD[b], Christoph C Kurmann[b], Patrik Michel MD[c], Steven D. Hajdu MD[d], Marc Ribo MD[e], Manuel Requena MD[e], Christian Maegerlein MD[f], Benjamin Friedrich MD[f], Vincent Costalat MD[g], Amel Benali MSc[g], Laurent Pierot MDh[i], Matthias Gawlitza MD[h], Joanna Schaafsma MD[i], Vitor Mendes Pereira MD[j], Jan Gralla MD[a]* and Urs Fischer MD[b]*

a University Institute of Diagnostic and Interventional Neuroradiology, University Hospital Bern, Inselspital, University of Bern, Bern, Switzerland
b Department of Neurology, University Hospital Bern, Inselspital, University of Bern, Bern, Switzerland
c Department of Neurology, CHUV Lausanne, Lausanne, Switzerland
d Department of Radiology, CHUV Lausanne, Lausanne, Switzerland
e Department of Neurology, Department of Neurology, Vall d'Hebron University Hospital, Barcelona, Spain.
f Department of Diagnostic and Interventional Neuroradiology, Klinikum rechts der Isar, Technical University Munich, Munich, Germany
g Department of Neuroradiology, CHU Montpellier, Montpellier, France
h Department of Neuroradiology, CHU Reims, Reims, France
i Department of Neurology, Toronto Western Hospital, Toronto, ON, Canada
10 Joint Department of Medical Imaging, Toronto Western Hospital, Toronto, ON, Canada
* These authors contributed equally

ONLINE SUPPLEMENT

Supplementary Tables

Supplementary Table 1

**Center**	N	Time period	LVO anterior circulation (ICA, M1, M2)	Lost to follow-up (mRS day 90)	ASPECTS available (LVO anterior circulation)	% ASPECTS ≤5 (LVO anterior circulation)	% MRI as initial imaging modality	% bridging IVT	ASPECTS grading	Admission and 24h NIHSS	mRS at 90 days	mTICI grading	EC approval	Responsible EC / Administrative institution
Inselspital Bern, University Hospital Bern, University of Bern, Bern, Switzerland	966	2010-2017	91.8% (888/966)	5.6% (54/966)	863/888	176/863 (20.4%)	50.6% (487/963)	41.2% (398/966)	Research fellow blinded to clinical data	Board certified stroke neurologists	Stroke neurologists on scheduled clinical visits. Structured telephone interviews if the patient was unable to attend (either by physician or mRS certified stroke nurse).	Operator-measured	Yes	Kantonale Ethik Kommission Bern
Toronto Western Hospital - University Health Network, University of Toronto, Toronto, Canada	60	2014-2017	88.3% (53/60)	0% (0/60)	53/53	1/53 (1.9%)	1.7% (1/60)	51.7% (31/60)	Prospective, by neuroradiologist	Board certified stroke neurologists	Clinical visits at the university hospital. For patients still in rehabilitation facilities, a mRS certified nurse schedules telephone interviews.	Operator-measured	Yes	IRB Toronto
Klinikum rechts der Isar, Technical University Munich, Munich, Germany	206	2009-2017	74.3% (153/206)	18.4% (38/206)	151/153	0/153 (0%)	2.4% (5/206)	55.8% (115/206)	Rerospective by neuroradiologist	Board certified stroke neurologists	mRS was evaluated either by face-to-face assessments (by stroke neurologists) or standardized telephone interviews (by certified study nurses).	Operator Measured	Yes	Ethikkommission der medizinischen Fakultät der Technischen Universität München
University Hospital Vall d'Hebron, Barcelona, Spain	418	2010-2017	85.9% (359/418)	20.1% (84/418)	319/359	4/319 (1.3%)	0% (0/491)	44.0% (184/418)	Prospective, by neurologist/neuroradiologist on call	Board certified stroke neurologists	Stroke neurologists on scheduled clinical visits. Structured telephone interviews if unable to attend.	Operator Measured	Yes	CEIC H. Vall d'Hebrond
CHUV, Lausanne University Hospital, Lausanne, Switzerland	139	2012-2017	124/139 (89.2%)	26.6% (37/139)	113/124	8/113 (7.1%)	0.1% (1/139)	71.9% (100/139)	Consensus stroke neurologist and neuroradiologist (not blinded)	Board certified stroke neurologists	mRs was assessed by Rankin-certified physicians at 3 months in the outpatient clinic, or alternatively through a structured telephone interview by Rankin-certified personnel.	Operator-measured	Yes	Commission Ethique de Recherche, Canton de Vaud
Montpellier CHU, University Hospital Montpellier, Montpellier, France	149	2015-2017	97.3% (145/149)	4.0% (6/149)	109/145	23/109 (21.1%)	82.1% (96/117)	56.4% (84/149)	Operator-measured	Board certified stroke neurologists	Stroke neurologists on scheduled clinical visits. Structured telephone interviews if unable to attend.	Operator-measured	(Yes)	Consent was waived owning to the retrospective design (favorable opinion by CNIL)
CHU Reims, University Hospital Reims, Reims, France	108	2013 - 2017	90.7% (98/108)	0% (0/108)	96/98	38/96 (39.6%)	92.6% (100/108)	67.6% (73/108)	Retrospective, certified neuroradiologist	Board certified stroke neurologists	Stroke physician on clinical visits at university hospital or remote outpatient center.	Retrospective, certified neuroradiologist	Yes*	IRB Reims University Hospital, Champagne-Ardenne University.
**Total**	**2046**													
	*Ethics committee votum was waived due to the entirely retrospective nature of data collection													

Supplementary Table II – Successful reperfusion on non-hemorrhagic neurological worsening with strata of IVT pretreatment (Direct thrombectomy vs Bridging)

		**No non-hemorrhagic neurological worsening (N=131)**	**Non-hemorrhagic neurological worsening (N=21)**	**OR**	**95%-CI**
**Direct thrombectomy (without IVT) (N=88)**	TICI0-2a (N=14)	64.3% (9/14)	35.7% (5/14)	0.218	0.058-0.814
	TICI2b-3 (N=74)	89.2% (66/74)	10.8% (8/74)		
**Bridging (with IVT) (N=64)**	TICI0-2a (N=7)	57.1% (4/7)	42.9% (3/7)	0.128	0.022-0.742
	TICI2b-3 (N=57)	91.2% (52/57)	8.8% (5/57)		
		Mantel-Haenszel Statistics:	Estimated (common) Odds Ratio	0.183	0.065-0.524
			Breslow-Day test for heterogeneity	P=0.634	

Supplementary Table III – Estimated frequency of non-hemorrhagic neurological worsening as calculated from the SITS – International Stroke Thrombolysis Register (ISTR) data on minor stroke treated with IVT

	**SITS-ISTR relative frequency of nh-NW**	**Frequency of occlusion patterns in the NIHSS ≤8 study cohort:**	**Frequency of occlusion patterns in patients with available data on nh-NW† (N=152)**	**Estimated % of nh-NW in study cohort**
**Intracranial ICA/Carotid-T or tandem**	30.0% (95%-CI 14.6-51.9%)	20.2%	17.8%	5.34% (17.8%* 30.0%)
**Other ICA**	16.7% (95%-CI 10.5-25.4)	1.0%	0%	0%
**M1**	9.3% (95%-CI 4.8-17.3%)	50.8%	50.7%	4.72% (50.7%* 9.3%)
**M2**	5.8% (95%-CI 3.0-11.1%)	28.0%	31.6%	1.83% (31.6%* 5.8%)
		**Total (estimated)**		**11.89 %**
		**Total (observed)**		**13.8 %** (21/131)
nh-NW, non-hemorrhagic neurological worsening, †patients with sICH were excluded				

Supplementary Figures

Supplementary Figure I – Distribution of admission NIHSS within the subpopulation of patients presenting with NIHSS<8 (N=193)

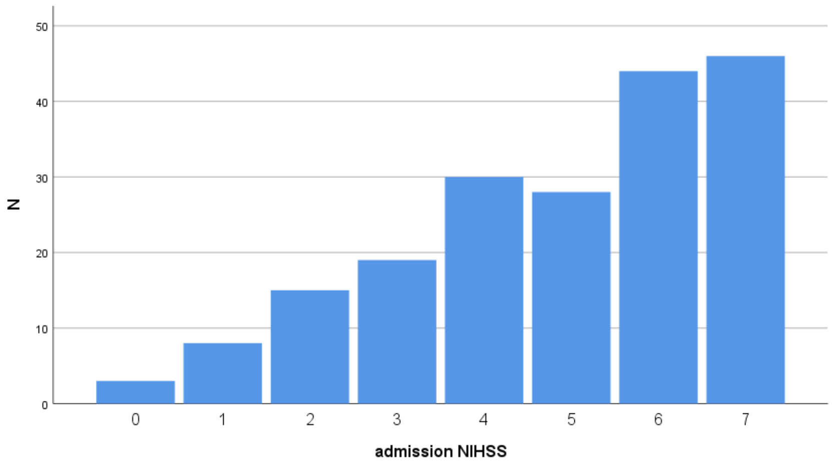

Median admission NIHSS in the subgroup of patients with NIHSS<8 was 5 (interquartile range 4-6). Most patients presented with NIHSS 6 and 7.

Supplementary Figure II – ΔNIHSS (admission- 24h) stratified according to TICI

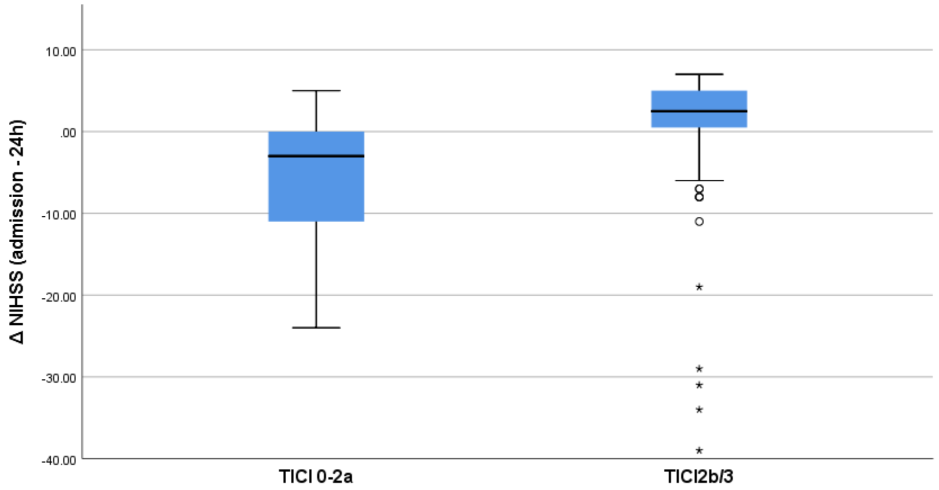

Median ΔNIHSS was -3 (interquartile range: -11 – 0) in non-successfully reperfused patients, while the NIHSS improved by 3 (interquartile range: 0-5) in successfully reperfused patients (P<.001).
